# Effect of Isolation Technique and Location on the Phenotype of Human Corneal Stroma-Derived Cells

**DOI:** 10.1155/2017/9275248

**Published:** 2017-10-29

**Authors:** Richárd Nagymihály, Zoltán Veréb, Andrea Facskó, Morten C. Moe, Goran Petrovski

**Affiliations:** ^1^Stem Cells and Eye Research Laboratory, Department of Ophthalmology, Faculty of Medicine, University of Szeged, Szeged, Hungary; ^2^Center for Eye Research, Department of Ophthalmology, Oslo University Hospital and University of Oslo, Oslo, Norway

## Abstract

**Purpose:**

To determine the effect of the isolation technique and location upon the phenotype of human corneal stroma-derived cells (CSCs).

**Methods:**

CSCs were isolated from the corneal stroma center and periphery using the explant or enzymatic digestion technique. The native tissue was stained for functional markers, while cultured cells were analysed by FACS. PCR was used to determine gene expression in the cultured versus native cells.

**Results:**

The native stroma was positive for *α*-actinin, ALDH1A1, CD31, CD34, Collagen I, and Vimentin. Cultured cells expressed CD73, CD90, CD105, CD51, Nestin, CD49a, CD49d, ABCG2, and CD47. PCR demonstrated a significant upregulation of ALDH1A1, AQP1, ITGB4, KLF4, CD31, CD34, and CXCR4 in the native tissue, while the expression of ABCG2, ITGAV, Nestin, CD73, CD90, CD105, and Vimentin were significantly higher in the cultured cells. GPC did not change.

**Conclusion:**

The study finds no significant difference between the phenotype of CSCs generated by the explant or enzymatic digestion technique from the center or periphery of the stroma. Isolation of the cells can be performed without regard to the location and isolation technique used for research. Cultivated CSCs undergo a complete surface marker and genotype profile change compared to the state in situ.

## 1. Introduction

The human corneal stroma is responsible for two-thirds of the refractive power of the eye and occupies 90% of the corneal thickness. When affected by disease or trauma, the homeostasis and thus transparency of the tissue is compromised. This is partially due to presence of local edema and activation of resident corneal cells—keratocytes. These quiescent cells assume a dendritic cell morphology *in vivo* and synthesize collagens and proteoglycans forming the backbone of the tissue [1]; when activated, keratocytes can transform into myofibroblasts, associated with scar formation and ultimately loss of corneal transparency [2, 3]. Treatment for such cases usually involves lamellar or penetrating keratoplasty, but due to donor scarcity worldwide, alternative sources are seeked upon, such as bioengineered or decellularised corneas or prostheses [4, 5].

Cells derived from the human corneal stroma have been shown to possess trilineage differentiation potential and presence or absence of specific markers (e.g., CD73^+^, CD90^+^, CD105^+^, CD34^−^, CD45^−^, CD14^−^, CD11b^−^, CD79*α*^−^, CD19^−^, and HLA-DR^−^), while adherence to plastic and exposure to serum render these cells equivalent to mesenchymal stem cells (MSCs) [6, 7]. Moreover, the immunosuppressive potential of CSCs has been demonstrated previously [8, 9], as well as their cell therapy potential in animal studies [10].

Establishment of CSC cultures is relatively easy. Nevertheless, many countries have access only to peripheral tissue remaining in unused corneal rings after keratoplasty. Use of enzymatic digestion to isolate CSCs out of the tightly packed collagen layers appears straightforward as well [11, 12], while the explant culture method of isolating pieces of corneal tissue to produce cells ex vivo has already been established. Many speculations yet remain about the true origin of the outgrowing cells from the corneal stroma ex vivo [9, 12]. Most likely, it is the resident cells of the corneal stroma that get activated after isolation and possibly different side populations become dominant, eventually generating a culture of cells displaying a fibroblastoid morphology [9, 13, 14].

Various types of culture media have also been assessed to elucidate the best possible conditions for induction of *in vitro* stem cell phenotype in these cells [15]; however, no comparison to date has been carried out to determine whether cells isolated from different locations (cornea center versus periphery) display any difference. The present study aims to establish cornea stromal cultures by explant and enzymatic digestion methods from central and peripheral parts of the cornea and compare their phenotypical and genotypical properties for future application of these cells in corneal research or cell therapy purposes.

## 2. Materials and Methods

### 2.1. Isolation Procedure and Cultivation

**Figure 1:**
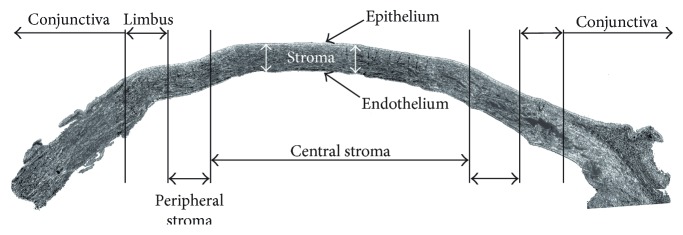
Anatomical features of the human cornea and sites of stromal cell isolation. Pieces of tissue were punched out from the indicated central and peripheral corneal regions by a surgical trephine (3 mm) for consequent digestion and/or culturing. The picture was taken by a phase contrast microscope and put together as an overlay (EVOS® FL microscope, Thermo Fisher Scientific).

Cadaveric tissue collection complied with the directive of the Helsinki Declaration and was approved by the National Medical Research Council (14387/2013/EKU-182/2013). Samples were obtained within 24 hours from death. Following disinfection by povidone iodine (Egis, Hungary) and rinsing with PBS of human bulbi, corneal buttons were dissected using scissors. The corneal epithelium, Descemet's membrane, and corneal endothelium were peeled off. To obtain equal-sized stromal explants, pieces of tissue measuring 3 mm in diameter were punched out from the corneal buttons with the help of a trephine from regions defined as the peripheral versus the central stroma, as shown in Figure 1. An equal number of punches were generated from the central versus peripheral regions in the same way described above and treated with 3 mg/mL (>125 CDU/mg) mixed collagenase solution (Sigma-Aldrich, St. Louis, MO, USA) for 3 hours at 37°C, with agitation.

Four types of CSC cultures were defined as central explant (CE) and peripheral explant (PE) versus central digested (CD) and peripheral digested (PD) from the same donors, referring to the location and presence or absence of enzymatic digestion, respectively. 24-well plates (Corning Costar, Sigma-Aldrich) were used to expand the cells. Dulbecco's Modified Eagle Medium (DMEM) (Sigma-Aldrich) supplemented with 10% Fetal bovine serum (FBS) (Sigma-Aldrich) and 1% penicillin-streptomycin (PS) (Sigma-Aldrich) was applied to the cells. Culture media was changed every alternate day. Cells up to passage 4 were used for the experiments.

### 2.2. Immunofluorescent Staining for Proliferation Marker Ki-67 in Cultured CSCs

CD, PD, CE, and PE were expanded in 24-well culture plates (Corning Costar). Cells were fixed in 4% paraformaldehyde (Sigma-Aldrich) and permeabilised using 0.1% Triton X-100 (Sigma-Aldrich). Bovine serum albumin (BSA) 1% (Sigma-Aldrich) diluted in phosphate-buffered saline (PBS) was applied as a blocking solution for 1 hour at room temperature. Samples were incubated with the primary Ki-67 (Sigma-Aldrich) antibody for 1 hour at room temperature. A phycoerythrin-conjugated secondary antibody was used to visualize the protein and finally, 4′,6-diamidino-2-phenylindole (DAPI, Sigma-Aldrich) counterstaining to stain the cell nuclei. Pictures were taken by an EVOS FL microscope (Thermo Fisher Scientific).

### 2.3. Immunofluorescent Staining of Native Corneal Tissue

For studying the protein expression in situ, corneal sections were prepared from paraffin-embedded tissues and stained for markers expressed by progenitor and/or stem cells (ABCG2, CXCR4, and Nestin). Proliferation- (Ki-67), function-related (ALDH1A1, Collagen I, and CD34), and MSC markers (CD73, CD90, CD105, and Vimentin), extracellular matrix and cell-adhesion components (Fibronectin, Collagen IV, and VE-Cadherin), and other molecules (*α*-actinin, ABCG5, and antifibroblast marker) were stained. More information about the antibodies used is shown in Table S1 available online at https://doi.org/10.1155/2017/9275248.

In brief, sections were deparaffinised and the nonspecific sites were blocked by 1% BSA (Sigma-Aldrich) for 1 hour at room temperature. Primary antibodies were applied overnight at 4°C. Following three times 5-minute wash by PBS containing 1% Tween-20 (PBST), Alexa Fluor 488 conjugated secondary antibodies were incubated on the sections for 1 hour at room temperature. DAPI counterstaining was performed to visualize the nuclei. Pictures were taken by a Zeiss Axio Observer Z1 (Carl Zeiss) microscope.

### 2.4. Fluorescence-Activated Cell Sorting (FACS)

CSCs were subcultured in 150 cm^2^ flasks (TPP, Sigma Aldrich) for FACS analyses. Cells were collected by trypsinisation (Hyclone, GE Healthcare Life Sciences, Logan, Utah, USA) for surface protein expression analyses. After centrifugation at 1000 RPM, for 10 minutes, the cells were resuspended in FACS buffer (0.05% Na-azide and 0.5% BSA in DPBS). Three-color staining—fluorescein-isothiocyanate, phycoerythrin, and allophycocyanin-conjugated primary antibodies against ABCG2, CD31, CD34, CD44, CD47, CD49a, CD49d, CD51, CD73, CD90, CD105, and Nestin—were applied for 30 minutes at 4°C. FACS Calibur cytometer (BD Biosciences, Immunocytometry Systems) was used to measure the samples. Finally, data were analysed by Flowing Software 2.5 (Perttu Terho, Turku Centre for Biotechnology, University of Turku, Finland) and FCS Express 6 (De Novo Software, California, USA). More information about the antibodies is provided in Table S2.

### 2.5. Reverse Transcription-Quantitative Polymerase Chain Reaction (RT-qPCR) Analysis

Native corneal stroma tissue free of epithelium and endothelium was collected from 3 donors. The tissue from the donors was homogenized and pooled. Total RNA was isolated by Qiazol reagent (Qiagen) and RNeasy mini kit (Qiagen) following the manufacturer's protocol. Similarly, total RNA was isolated from cultured cells separately for the 4 defined conditions by the RNeasy mini kit and pooled from 3 donors.

Nucleic acid concentrations were determined by a NanoDrop spectrophotometer (Thermo Fisher Scientific). Random hexamers and Superscript III reverse transcriptase (Life Technologies, Waltham, MA, USA) were used to transcribe 1 *μ*g RNA into cDNA. StepOnePlus RT-PCR system (Applied Biosystems) and Taqman Gene Expression assays were used to determine relative gene expression levels. Genes *ALDH1A1* (Hs00605167_g1), *ABCG2* (Hs01053790_m1), *AQP1* (Hs01028916_m1), *CD31* (Hs01065279_m1), *CD34* (Hs00990732_m1), *CD73* (Hs01573922_m1), *CD90*/*THY1* (Hs00174816_m1), *CXCR4* (Hs00607978_s1), *ENG* (*CD105)* (Hs00923996_m1), *GPC4* (Hs00155059_m1), *ITGAV* (Hs00233808_m1), *ITGB4* (Hs00236216_m1), *KLF4* (Hs00358836_m1), *Nestin* (Hs00707120_s1), and *Vimentin* (Hs 00185584_m1) were used for the analyses. 10 minutes at 95°C, then 40 cycles at 95°C (15 s) and 60°C for 1 minute was set for the measurements. Analysis of the data was done by the 2^−ΔΔCt^ method. Fold change (relative quantity) was determined relative to the expression level of the native stromal tissue. *18S RNA* (Hs03003630_g1) was used as a housekeeping gene. All samples were run in triplicates.

### 2.6. Statistical Analysis

One-way ANOVA and Student's *t*-test were applied to reveal statistical differences between different groups. Significance level was set to 0.95. *p* values less than 0.05 (^∗^*p* < 0.05; ^∗∗^*p* < 0.01) were considered significant.

## 3. Results

### 3.1. Cell Morphology and Proliferative Activity

**Figure 2:**
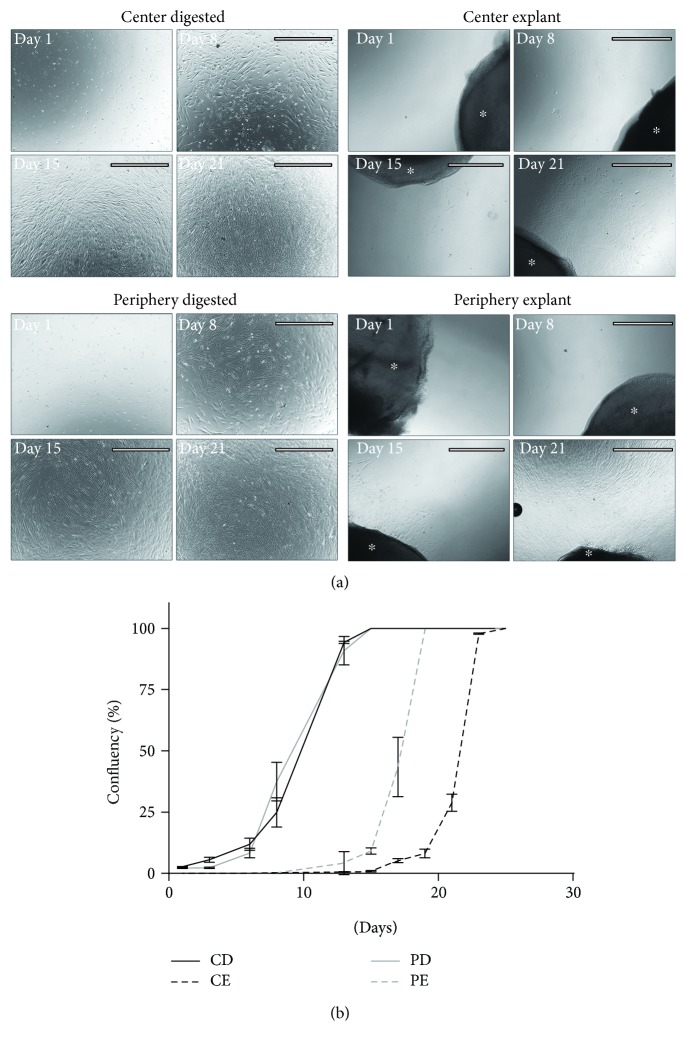
Phase contrast images of corneal stroma cell cultures and respective growth rates. Pictures show days 1, 8, 15, and 21 of cultivation for cells obtained from the central and peripheral regions of the stroma by enzymatic digestion and explant techniques (a). The scale bars represent 1000 *μ*m. The explanted tissue is marked by an asterisk (^∗^). Confluency of the cultured cells % ± SD was determined by ImageJ measurements for each isolation technique (*n* = 3), and the points were plotted accordingly for the given values on the respective days (b).

Cultures established by the enzymatic method (CD, PD) yielded CSCs immediately, and these cells proliferated fast (Figure 2(a)), reaching confluence within 10–12 days (Figure 2(b)). The explant cultures from the central and peripheral regions (CE, PE) showed no microscopically observable proliferative activity up until days 12–14, when the CSCs started actually migrating and proliferating around the edges of the explants (Figure 2(a)). The explant cultures reached confluency by days 20–25 after isolation (Figure 2(b)). No apparent morphological differences were observed between the CSCs produced from different locations (center versus periphery) and isolation technique (explant versus enzymatic digestion).

**Figure 3:**
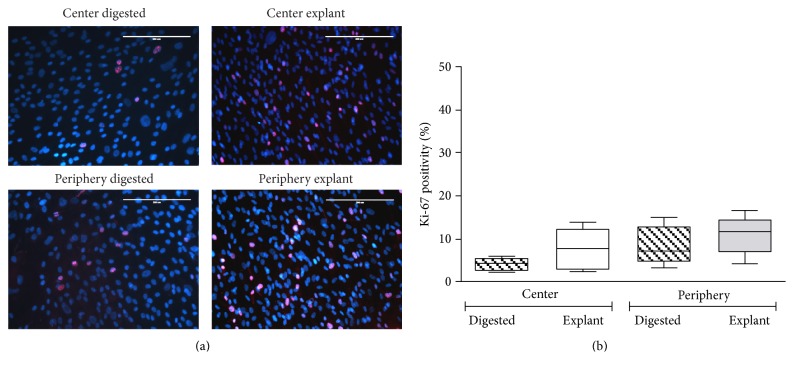
Immunofluorescent staining of nuclear Ki-67 in cultured cells. CSCs obtained from either the central or peripheral corneal stroma by digestion and explant techniques have been cultured for 21–30 days, respectively, and stained for proliferation marker Ki-67. The proliferation marker is shown in red with DAPI counterstaining (a). Relative quantity of Ki-67-positive cells (%) ± SD is shown (b) (*p* = 0.43). The scale bars represent 200 *μ*m.

Ki-67 staining revealed a strong proliferative capacity of the cells in both the explant and the digested cultures isolated from the central and peripheral regions of the corneal stroma (Figure 3(a)). From all stained cells, 4.21 ± 1.53%, 7.87 ± 4.73%, 8.60 ± 4.58%, and 10.95 ± 4.42% were positive for Ki-67 (Figure 3(b)) for the four different conditions: CD, CE, PD, and PE, respectively (*p* = 0.43).

### 3.2. Immunofluorescent Staining of the Native Corneal Stroma

**Figure 4:**
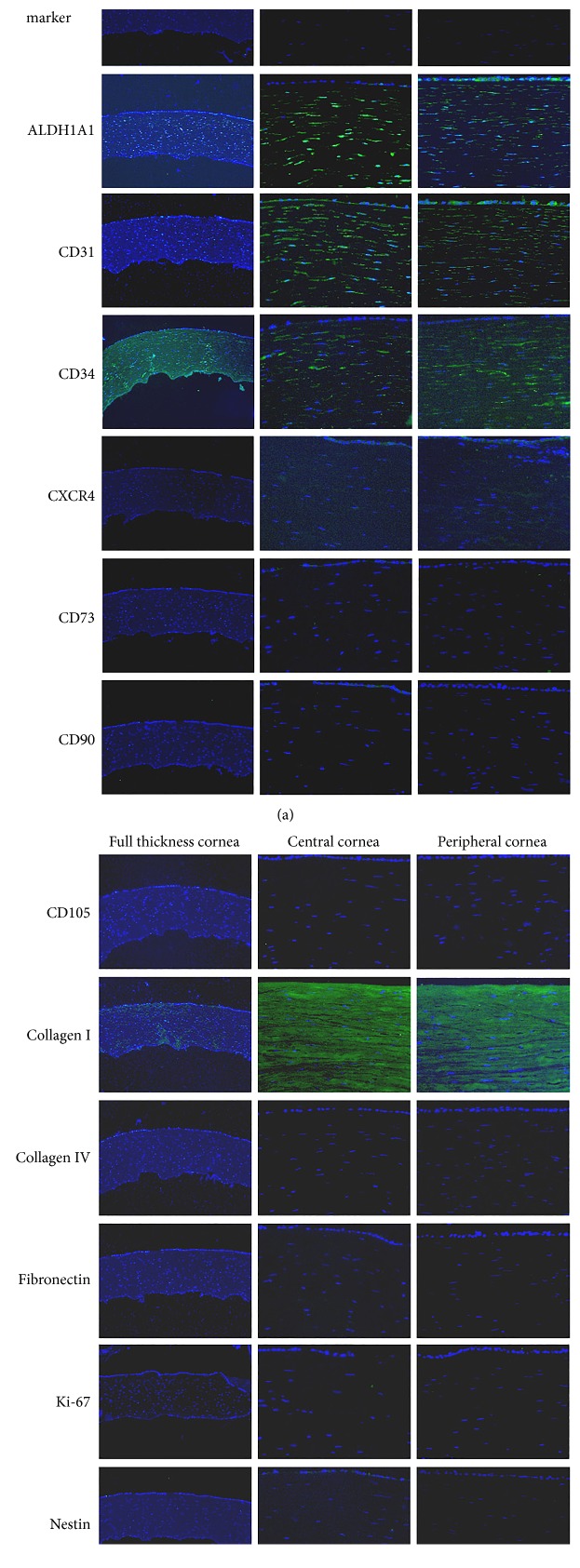
Immunofluorescent staining of normal human full thickness corneal sections. Images were acquired at 10x (left column) and 40x (middle and right columns) magnifications for each marker. Proteins and markers were stained by Alexa Fluor 488 conjugated secondary antibodies (green). DAPI (blue) counterstaining was applied to visualize the cell nuclei.

In order to first demonstrate the differential expression patterns of cultured versus resident cells of the cornea, the expression of markers for stemness, mesenchymal, epithelial, endothelial cell origin, and extracellular matrix components and adhesion proteins was carried out in the native cornea (Figure 4 and Table 1, for a summary of the findings). The anterior stroma is defined as the first 1/3 of the thickness of the cornea proximal to the corneal epithelium, while posterior stroma is defined as the 2/3 thickness proximal to the corneal endothelium (Figure S1).

Expression of ABCG2 and ABCG5 could not be detected in situ in any of the corneal layers or regions. Strong and similar staining for ALDH1A1 and *α*-actinin was observed in the central and peripheral regions of the stroma. The presence of CD34 was confirmed by a strong signal coming from all parts of the stroma in situ and CD31 was detected as well. (Figure 4).

The expression of the major stromal component, Collagen I, showed strong positivity, while absence of Collagen IV could be detected throughout the stroma. The triad of MSC markers: CD73, CD90, and CD105 was negative throughout the native cornea, and markers like Ki-67, CXCR4, and Nestin could not be detected in the tissue either. The presence of Vimentin could be confirmed in the stroma, while Fibronectin appeared to be negative in the native corneal stroma. The expression of an antireticulocyte, fibroblast marker, and VE-cadherin was also found negative in this tissue (Figure 4).

### 3.3. Surface Protein Expression Profile of Cultured CSCs

**Figure 5:**
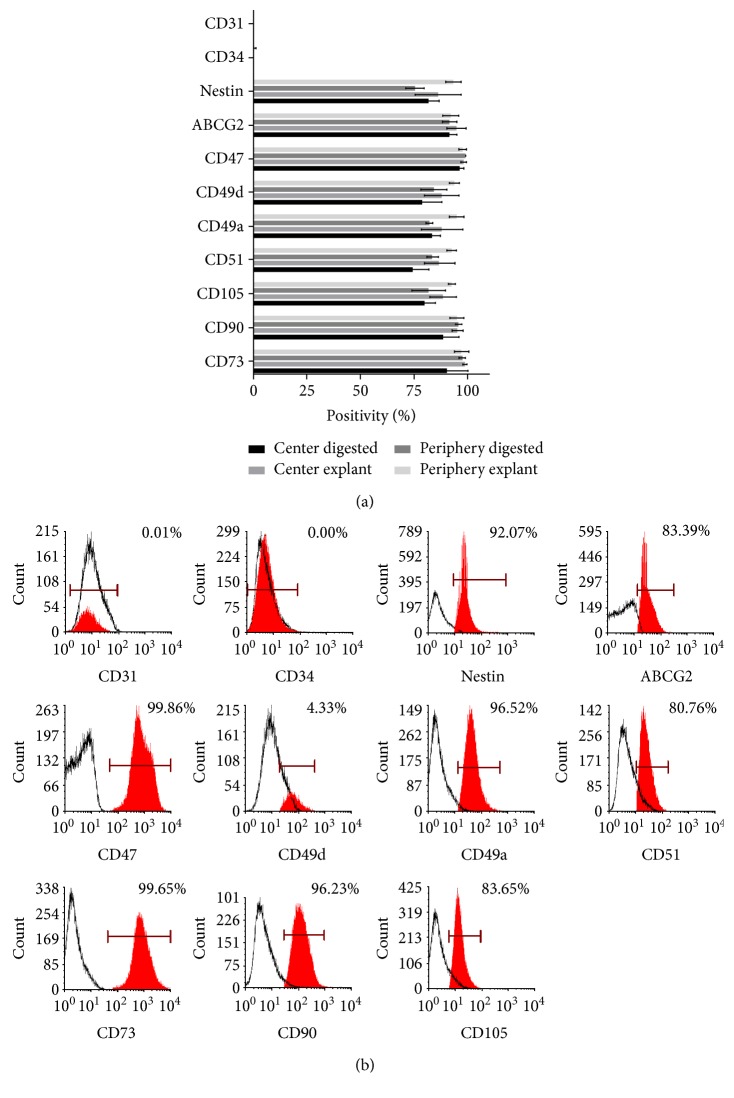
Percent of positive cells for given surface markers in the four different conditions ± SD is shown (a) (*n* = 3). Representative histograms of the FACS analysis, showing a PE donor, with the respective isotype controls, depicted in white color, with the stated antibodies in red, after Overton histogram subtraction (% of positive cells is shown) (b).

FACS analyses revealed a high expression of MSC markers: CD73, CD90, and CD105, with no significant difference among the culture conditions (Figure 5 and Table S3). Adhesion molecules CD51, CD49a, CD49d, and integrin-related CD47 showed an increased expression, with no significant difference being detected between the conditions. Putative stem cell markers ABCG2 and Nestin were positive, too, while CD34 and CD31 were negative. Statistical analyses revealed no significant difference in the expression of the latter proteins when comparing the various isolation conditions used.

### 3.4. Gene Expression Pattern in Cultured CSCs and Native Corneal Stroma

**Figure 6:**
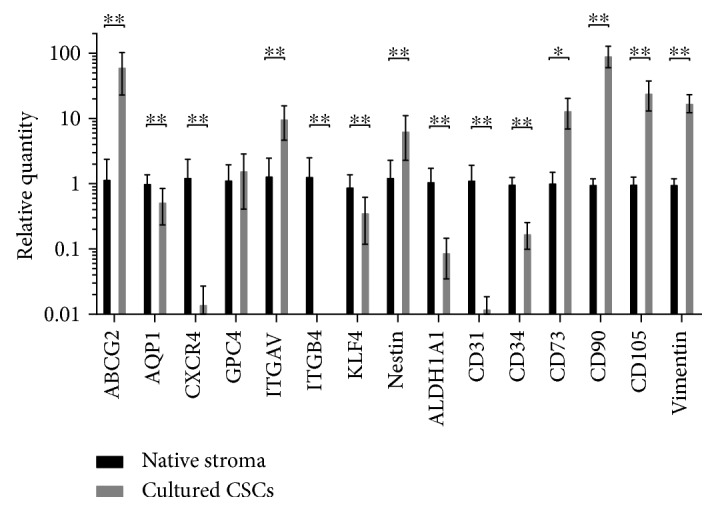
Gene expression profile of the native corneal stroma compared to cultured CSCs. Relative quantities are shown on a logarithmic scale, in the native and pooled cultured cells from the different isolation/cultivation methods; mean relative quantity (RQ) ± standard deviation (SD) is represented. Significance values are depicted as ^∗^*p* < 0.05 and ^∗∗^*p* < 0.01. 18S RNA was used as a housekeeping gene, with the expression levels of the cultured CSCs compared to that of the native tissue (*n* = 3). The RNA from the native tissue was prepared by mincing the stromal tissue from 3 donors into pieces, followed by extraction of total RNA.

CD73, CD90, and CD105/Endoglin were significantly expressed higher in the cultured CSCs compared to the native stroma (14-, 95-, and 25-fold; *p* = 0.01,*p* < 0.01, and *p* < 0.01, resp.). Furthermore, no difference could be detected in the expression of the latter genes in the different regions isolated by the two methods. Expression of Vimentin appeared to be 18-fold higher in the cultured CSCs compared to the native stroma (*p* < 0.01) with no difference between the various culture conditions. The expression of CD34 was significantly downregulated in cultured cells, as much as 5-fold, when compared to the expression found in the native tissue (*p* < 0.01). CD31 was significantly downregulated in cultured CSCs versus native cells (*p* < 0.01). Significantly lower expression of ALDH1A1 could be detected in the culture conditions compared to the native corneal stroma as well (*p* < 0.01) (Figure 6).

ABCG2 was expressed 60-fold more in primary CSCs compared to the native stromal cells (*p* < 0.01). Significantly lower expressions of AQP1, CXCR4, ITGB4, and KLF4 were detected in the cultured CSCs compared to that of the native tissue (*p* < 0.01, *p* < 0.01, *p* < 0.01, and *p* < 0.01, resp.), while ITGAV and Nestin were significantly upregulated in the cultures (*p* < 0.01, *p* < 0.01). GPC4 expression was found to be unaffected by culturing (*p* = 0.36).

## 4. Discussion

Cells derived from the corneal stroma can be a good source for corneal research, drug testing, and future cell therapy purposes in the eye or other organs [9, 16]. CSCs derived from explants from the central part of the human corneal stroma have been extensively characterized by us recently [6]. These cells display MSC-like properties *in vitro*, including trilineage differentiation potential and immunosuppressive characteristics. Such features, however, appear not to be characteristic of the resident cells *in vivo*. It is still debated whether cells obtained from the stroma stem-like cells are actually stromal keratocytes that undergo morphological and functional changes or a different small progenitor population existent *in vivo*, which gets activated ex vivo.

The shortage of donor corneas worldwide [17] and the availability of corneal rings remaining after keratoplasty in many research groups worldwide justify a comparison and clarification which of the different sources or techniques for isolating corneal stromal cells can be used. Most of the available tissue following surgery contains stroma from the periphery, such as that remaining after penetrating keratoplasty, DMEK, or DSAEK procedures.

Immunostaining of the native corneal tissue revealed no significant difference in the expression of previously described markers in the central versus peripheral parts of the cornea. The native corneal stroma showed no expression of the putative stem cell marker—the efflux protein ABCG2—while a strong staining was observed in the cultures from both the central and peripheral regions produced by both techniques of isolation (explant versus enzymatic). This difference in the expression found in the cultured CSCs versus the native cells could also be confirmed at the gene expression level. Upregulation of ABCG2 may result in a stronger resistance of cultured cells to externally applied therapy (e.g., chemotherapy), while cancerous cells have been known to exploit use of such molecules to survive harsh conditions [18, 19]. A population of murine adult stem cells stained by Hoechst 33342 has been shown to discharge the dye through ABCG2 and could be inhibited by verapamil [20]. Similarly, a side population has been identified in the human limbal epithelium [21]. No expression of another member of the ATP-binding cassette transporter superfamily, such as ABCG5, could be found in the native cornea.

Expression of ALDH1A1, a corneal crystalline, is essential for the maintenance of transparency, downregulation of which is associated with corneal haze [22]. Strong staining was observed in the corneal stroma, which was equally distributed along the central and peripheral regions of the cornea. Interestingly, its expression was significantly downregulated in the culture conditions compared to the native tissue.

All layers of the cornea expressed *α*-actinin, including the stroma. Since keratocytes express this protein, the marker should not be used alone for excluding presence of fibroblastic cells *in vivo* or ex vivo [23].

CD31 is usually expressed by vascular endothelial cells and is likely to be involved in leukocyte migration. The role of CD31 in the attraction and adhesion of polymorphonuclear cells in corneal wound healing has been demonstrated before [24]; however, its role in an undamaged and purely avascular tissue such as the native cornea remains unclear. Interestingly, the ex vivo cultured CSCs elicited no CD31 positivity by the means of isolation applied here. The RT-qPCR analysis confirmed a statistically significant downregulation of CD31 in the cultured versus the native cells.

Little is known about the pleiotropic functions of CD34, which is often referred to as the marker of hematopoietic progenitors, while evidence suggests this marker to likely function in immunological processes, such as regulating migration and mobility of eosinophil granulocytes and dendritic cells, as demonstrated in animal knockout experiments [25]. Several studies have demonstrated the presence of CD34 in keratocytes and its loss over the cultivation time ex vivo [16, 26]. Strong staining of the corneal stroma could indeed be detected in the native corneas, which was not the case in the cultured cells. Gene expression analysis further supported this finding, and a 5-fold decrease in the expression of CD34 was observed in the cultured CSCs compared to the native stroma. This is also in line with the findings of others, which still leaves the function of this protein to be further elucidated in the cornea [16]. Aquaporin-1 is an important factor in keratocyte migration during wound healing, *in vivo*, which is downregulated, yet still expressed *in vitro* in an animal model [27].

The triad of MSC markers—CD73, CD90, and CD105—was found to be negative in situ, in contrast to the strong positivity observed in all cultured cells ex vivo. The same results were found when comparing the gene expression of the cultured versus native cells. These findings are supported by recent findings in another independent study [28], which shows the strong potential corneal stroma cells have, as well as their ability to get activated upon cultivation ex vivo. Gaining expression of the latter three proteins, together with a loss of CD34 during ex vivo cultivation, is what renders CSCs to have MSC-like phenotype, according to the International Society for Cellular Therapy (ISCT). Other types of MSCs of different origin demonstrate similar surface phenotype characteristics (high expression of CD90 and CD105) [29–32]. CD90 is believed to be involved in the cellular adhesion to the matrix and other cells, inflammation, fibrosis, migration, and tumour growth, [33] while CD73 is likely responsible for the immunosuppressive role of MSCs [34]. This feature has been demonstrated by us recently, as well as [6]. These molecules are retained over long-term and multiple passages on the cells, as demonstrated by us and others equally [35].

Characterizing the extracellular matrix which makes the backbone of the cornea is also very important to elucidate the difference between the corneal stroma cells *in vivo*, while in their niche, and ex vivo. Abundance of the major component of the corneal stroma, Collagen I, was indeed found throughout the native stroma, while Collagen IV was absent. Integrin *α*V (CD51) was found to be expressed on cultured cells and an upregulation was observed at a gene level, while Integrin *β*4 was significantly downregulated ex vivo compared to in situ.

These findings further strengthen the cultured cells respond to a change in the environmental niche surrounding them, which is likely compensated by deposition of de novo synthesized collagen ex vivo (data not shown).

Fibronectin was also not present in the native, intact corneas, which confirms the corneal wound healing properties of this extracellular matrix component. Deposits of Fibronectin have been shown to appear in the epithelium and stroma soon after penetrating trauma, although it disappears over the course of two weeks [36].

The presence of Ki-67 could not be detected in the native corneal sections either. This further confirms there was no trauma affecting the control, native epithelium, or the other layers of the cornea, thus indicating presence of induced cell proliferation, although both explant and enzymatic technique generated cultures from the different corneal regions displayed actively proliferating Ki-67-positive cells.

The mesenchymal marker Vimentin was found to be expressed in the stroma. This is in line with the findings of others. Vimentin has also been shown in knockdown studies to cause development of corneal haze [37]. The neural stemness marker Nestin could not be detected in the native corneal stroma. This protein is known to be expressed in spherical cultures generated from murine corneas, while putative precursor Nestin-positive population has been shown previously in the peripheral cornea of rabbits [38, 39]. Nestin was found to be upregulated in the cultured cells when compared to the native tissue. The protein is expressed in proliferating cells and is believed to have a role in the reorganization of intermediate filaments.

KLF4, an important stemness marker, has been associated with tissues exposed to the outside world [40]. The cornea is the first and most important mechanical barrier of the eye. We hereby show a decreased gene expression of KLF4 in cultured CSCs, compared to the native stroma. The putative stem cell migratory/homing marker CXCR4 [41, 42] was not detected in situ by immunostaining, while low levels were detected by PCR. Others reported similar, low amounts of the functional CXCR4 in cultured MSCs, but blocking the molecule has led to a decreased homing response to bone marrow [43]. CXCR4 has also been implicated in the invasion of malignant cells and epithelial-mesenchymal transition (EMT) [44].

Altogether, a general upregulation of stemness and mesenchymal cell markers was observed in the ex vivo cultivated CSCs, with a downregulation of function-related molecules, which should all be considered when treating such cells as MSCs. The potential of corneal stromal cells has been demonstrated numerous times in animal studies before; however, it seems like certain compromises should be made when using such cells as part of *in vitro* study models, due to the striking differences *in vivo* and *in vitro*.

The present study shows no phenotypic or genotypic difference between CSCs produced by the digestion or explant methods from the central or peripheral regions of the cornea. However, the gene expression and protein profile of native corneal stroma cells compared to ex vivo expanded CSCs shows that the latter likely adapt from an *in vivo* extracellular matrix niche to an adopted environment and presence of serum in the culture medium. Such a change in the expression profile shows how dynamic corneal stroma cells can be to the environmental niche and likely to wounds or inflammation on the surface of the eye and intracorneally. It remains to be further examined whether such changes are reversible, or if it is beneficial for the cells to change their gene and protein expression if used for treatment of corneal, eye, or other conditions in human cell therapy. Most likely, a future biopsy taken from any part of a healthy live donor, despite the procedure being invasive, could yield viable, expanding populations of stromal cells exhibiting mesenchymal stem cell-like properties.

## Supplementary Material

Figure S1. Definition of the anterior (towards corneal epithelium) and posterior (towards corneal endothelium) corneal stroma. The corneal sections have been stained for nuclei with DAPI (A) and with H&E (B). Table S1. Antibodies used for immunofluorescent staining. Table S2. Antibodies used for the FACS analyses of cultured CSCs. Table S3. Surface marker FACS analyses of the CSCs cultivated under different conditions. Positive cells ± SD are shown (*n*=3).

## Figures and Tables

**Table 1:** Distribution of corneal stroma markers in the various corneal regions.

Marker	Peripheral stroma	Central stroma	Anterior stroma	Posterior stroma
ABCG2	−	−	−	−
ABCG5	−	−	−	−
ALDH1A1	++	++	++	+
*α*-Actinin	++	++	++	+
CD31	+	+	+	+
CD34	++	++	++	++
CD73	−	−	−	−
CD90	−	−	−	−
CD105	−	−	−	−
Collagen I	++	++	++	++
Collagen IV	−	−	−	−
CXCR4	−	−	−	−
Fibroblast marker	−	−	−	−
Fibronectin	−	−	−	−
Ki-67	−	−	−	−
Nestin	−	−	−	−
VE-Cadherin	−	−	−	−
Vimentin	++	++	++	++

“−” stands for no staining, “+” for a medium intensity signal, and “++” for a strong staining.
