# Supplementary material for: Effect of Isolation Technique and Location on the Phenotype of Human Corneal Stroma-Derived Cells
Source: Stem Cells Int. 2017 Oct 29;2017:9275248. doi: 10.1155/2017/9275248 (PMC5682086; doi:10.1155/2017/9275248)
Supplement: Supplementary file 1 — Figure S1. Definition of the anterior (towards corneal epithelium) and posterior (towards corneal endothelium) corneal stroma. The corneal sections have been stained for nuclei with DAPI (A) and with H&E (B). Table S1. Antibodies used for immunofluorescent staining. Table S2. Antibodies used for the FACS analyses of cultured CSCs. Table S3. Surface marker FACS analyses of the CSCs cultivated under different conditions. Positive cells ± SD are shown (n=3). [file 9275248.f1.pdf]

Supplementary material

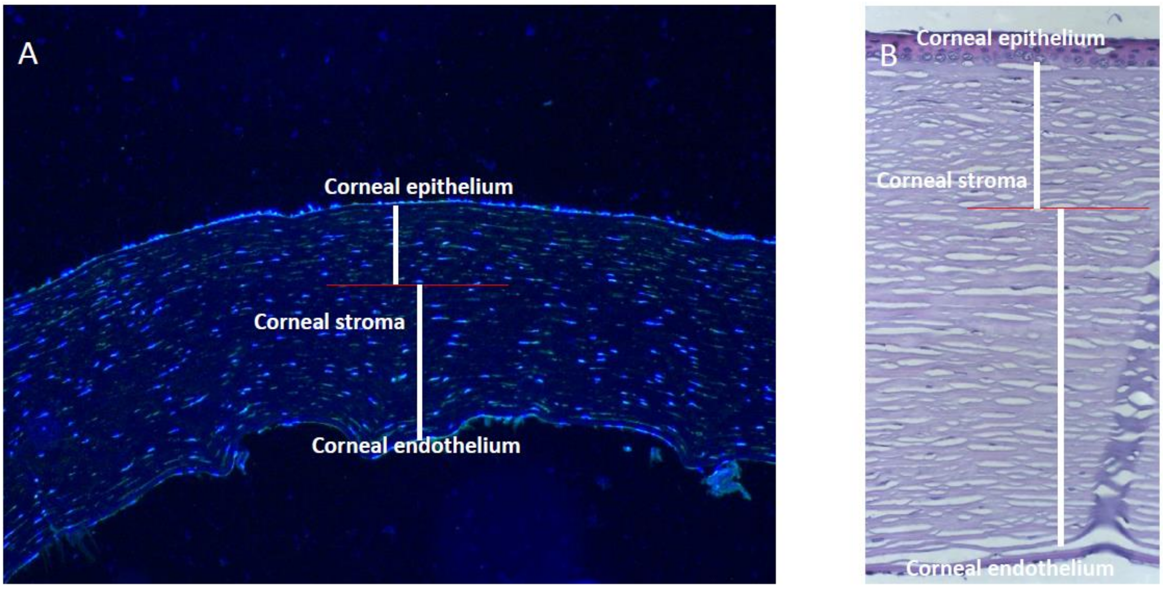

Figure S1. Definition of the anterior (towards corneal epithelium) and posterior (towards corneal endothelium) corneal stroma. The corneal sections have been stained for nuclei with DAPI (A) and with H&E (B).

Table S1. Antibodies used for immunofluorescent staining

Marker	Clone	Cat. No.	Manufacturer
ABCG2	BXP-21	B7059	Sigma-Aldrich
ABCG5	Not available	PAB20881	Abnova
α-Actinin	BM-75.2	A5044	Sigma
Anti-fibroblast marker	AS02	Cp28	Calbiochem
ALDH1A1	Not available	Ab23375	Abcam
CD31	M20	Sc1506	Santa Cruz
CD34	BI-3C5	Sc19621	Santa Cruz
CD73	IE9	Sc32299	Santa Cruz
CD90	H-110	Sc9163	Santa Cruz
CD105	H-300	Sc20632	Santa Cruz
CXCR4	UMB2	Ab124824	Abcam
Collagen I	Not available	Ab34710	Abcam
Collagen IV	CIV22	Ms-747-s	Thermo Scientific
Fibronectin	FN30.8	M010	Takara Bio ink
Ki-67	SP6	RM-9106-S	Thermo Scientific
Nestin	10c2	Sc23927	Santa Cruz
VE- Cadherin	BV9	Ab7047	Abcam
Vimentin	Sp20	RM-9120-s	Thermo Scientific

Table S2. Antibodies used for the FACS analyses of cultured CSCs.

Protein	Clone	Cat. No.	Manufacturer
ABCG2	5D3	332020	Biolegend
CD31	WM59	303106	Biolegend
CD34	581	343504	Biolegend
CD44	BJ18	338804	Biolegend
CD47	472603	FAB4670A	R&D Systems
CD49a	TS2/7	328304	Biolegend
CD49d	9F10	304304	Biolegend
CD51	NKI-M9	327908	Biolegend
CD73	AD2	344004	Biolegend
CD90	5E10	328108	Biolegend
CD105	43A3	323206	Biolegend
Nestin	196908	IC1259P	R&D Systems

Table S3. Surface marker FACS analyses of the CSCs cultivated under different conditions. Positive cells ± SD are shown (n=3).

Surface marker	Central digested (%)	Central explant (%)	Peripheral digested (%)	Peripheral explant (%)
CD73	90.39±9.73	98.71±0.97	97.45±1.49	97.18±3.31
CD90	88.70±7.27	95.33±2.51	95.78±1.52	94.96±3.24
CD105	79.88±5.00	88.55±6.14	81.81±7.85	92.64±1.62
CD51	74.55±7.30	86.94±7.17	83.68±2.78	92.49±2.15
CD49a	83.24±4.05	88.09±9.58	82.16±1.57	94.96±3.37
CD49d	78.98±8.94	87.97±8.02	84.31±6.01	93.79±2.33
CD47	96.30±1.83	98.07±1.30	99.28±0.09	97.71±1.69
ABCG2	91.45±3.42	94.82±4.49	91.56±3.37	92.11±3.65
Nestin	81.64±5.15	86.28±10.72	75.47±4.38	93.42±3.53
CD34	0.02±0.02	0.00±0.00	0.19±0.11	0.67±0.67
CD31	0.13 ± 0.13	0.00 ± 0.00	0.00 ± 0.00	0.00 ± 0.00
