# Supplementary material for: Effect of Isolation Technique and Location on the Phenotype of Human Corneal Stroma-Derived Cells
Source: Stem Cells Int. 2017 Oct 29;2017:9275248. doi: 10.1155/2017/9275248 (PMC5682086; doi:10.1155/2017/9275248)
Supplement: Supplementary file 2 [file 9275248.f2.docx]

# Supplementary material

**
Figure S1. Definition of the anterior (towards corneal epithelium) and posterior (towards corneal endothelium) corneal stroma.** The corneal sections have been stained for nuclei with DAPI (A) and with H&E (B)**.**

**Table S1**. Antibodies used for immunofluorescent staining

| **Marker** | **Clone** | **Cat. No.** | **Manufacturer** |
| --- | --- | --- | --- |
| ABCG2 | BXP-21 | B7059 | Sigma-Aldrich |
| ABCG5 | Not available | PAB20881 | Abnova |
| α-Actinin | BM-75.2 | A5044 | Sigma |
| Anti-fibroblast marker | AS02 | Cp28 | Calbiochem |
| ALDH1A1 | Not available | Ab23375 | Abcam |
| CD31 | M20 | Sc1506 | Santa Cruz |
| CD34 | BI-3C5 | Sc19621 | Santa Cruz |
| CD73 | IE9 | Sc32299 | Santa Cruz |
| CD90 | H-110 | Sc9163 | Santa Cruz |
| CD105 | H-300 | Sc20632 | Santa Cruz |
| CXCR4 | UMB2 | Ab124824 | Abcam |
| Collagen I | Not available | Ab34710 | Abcam |
| Collagen IV | CIV22 | Ms-747-s | Thermo Scientific |
| Fibronectin | FN30.8 | M010 | Takara Bio ink |
| Ki-67 | SP6 | RM-9106-S | Thermo Scientific |
| Nestin | 10c2 | Sc23927 | Santa Cruz |
| VE- Cadherin | BV9 | Ab7047 | Abcam |
| Vimentin | Sp20 | RM-9120-s | Thermo Scientific |

**Table S2**. Antibodies used for the FACS analyses of cultured CSCs.

| **Protein** | **Clone** | **Cat. No.** | **Manufacturer** |
| --- | --- | --- | --- |
| ABCG2 | 5D3 | 332020 | Biolegend |
| CD31 | WM59 | 303106 | Biolegend |
| CD34 | 581 | 343504 | Biolegend |
| CD44 | BJ18 | 338804 | Biolegend |
| CD47 | 472603 | FAB4670A | R&D Systems |
| CD49a | TS2/7 | 328304 | Biolegend |
| CD49d | 9F10 | 304304 | Biolegend |
| CD51 | NKI-M9 | 327908 | Biolegend |
| CD73 | AD2 | 344004 | Biolegend |
| CD90 | 5E10 | 328108 | Biolegend |
| CD105 | 43A3 | 323206 | Biolegend |
| Nestin | 196908 | IC1259P | R&D Systems |

**Table S3.** Surface marker FACS analyses of the CSCs cultivated under different conditions. Positive cells ± SD are shown (n=3).

| Surface marker | Central digested (%) | Central explant (%) | Peripheral digested (%) | Peripheral explant (%) |
| --- | --- | --- | --- | --- |
| CD73 | 90.39±9.73 | 98.71±0.97 | 97.45±1.49 | 97.18±3.31 |
| CD90 | 88.70±7.27 | 95.33±2.51 | 95.78±1.52 | 94.96±3.24 |
| CD105 | 79.88±5.00 | 88.55±6.14 | 81.81±7.85 | 92.64±1.62 |
| CD51 | 74.55±7.30 | 86.94±7.17 | 83.68±2.78 | 92.49±2.15 |
| CD49a | 83.24±4.05 | 88.09±9.58 | 82.16±1.57 | 94.96±3.37 |
| CD49d | 78.98±8.94 | 87.97±8.02 | 84.31±6.01 | 93.79±2.33 |
| CD47 | 96.30±1.83 | 98.07±1.30 | 99.28±0.09 | 97.71±1.69 |
| ABCG2 | 91.45±3.42 | 94.82±4.49 | 91.56±3.37 | 92.11±3.65 |
| Nestin | 81.64±5.15 | 86.28±10.72 | 75.47±4.38 | 93.42±3.53 |
| CD34 | 0.02±0.02 | 0.00±0.00 | 0.19±0.11 | 0.67±0.67 |
| CD31 | 0.13 ± 0.13 | 0.00 ± 0.00 | 0.00 ± 0.00 | 0.00 ± 0.00 |
